# Gender differences in quality of life and the course of schizophrenia: national study

**DOI:** 10.1192/bjo.2022.3

**Published:** 2022-02-01

**Authors:** Anat Rotstein, Efrat Shadmi, David Roe, Marc Gelkopf, Stephen Z. Levine

**Affiliations:** Department of Community Mental Health, Faculty of Social Welfare and Health Sciences, University of Haifa, Israel; Cheryl Spencer Department of Nursing, Faculty of Social Welfare and Health Sciences, University of Haifa, Israel; Department of Community Mental Health, Faculty of Social Welfare and Health Sciences, University of Haifa, Israel; Department of Community Mental Health, Faculty of Social Welfare and Health Sciences, University of Haifa, Israel; Department of Community Mental Health, Faculty of Social Welfare and Health Sciences, University of Haifa, Israel

**Keywords:** Epidemiology, males, females, Manchester Short Assessment of Quality of Life, national registry data

## Abstract

**Background:**

Evidence from various sources suggests that females with schizophrenia tend to report lower quality of life than males with schizophrenia despite having a less severe course of the disorder. However, studies have not examined this directly.

**Aims:**

To examine gender differences in the association between quality of life and the risk of subsequent psychiatric hospital admissions in a national sample with schizophrenia.

**Method:**

The sample consisted of 989 (60.90%) males and 635 (39.10%) females with an ICD-10 diagnosis of schizophrenia. Quality of life was assessed and scored using the Manchester Short Assessment of Quality of Life. The course of schizophrenia was assessed from the number of psychiatric hospital admissions. Participants completed the quality of life assessment and were then followed up for 18-months for subsequent psychiatric admissions. Hazard ratios (HR) from Cox proportional hazards regression models were estimated unadjusted and adjusted for covariates (age at schizophrenia onset and birth year). Analyses were computed for males and females separately, as well as for the entire cohort.

**Results:**

A subsample of 93 males and 55 females was admitted to a psychiatric hospital during follow-up. Higher quality of life scores were significantly (*P* < 0.05) associated with a reduced risk of subsequent admissions among males (unadjusted: HR = 0.96, 95% CI 0.93–0.99; adjusted HR = 0.96, 95% CI 0.93–0.99) but not among females (unadjusted: HR = 0.97, 95% CI 0.93–1.02; adjusted HR = 0.97, 95% CI 0.93–1.02).

**Conclusions:**

Quality of life in schizophrenia is a gender-specific construct and should be considered as such in clinical practice and future research.

Schizophrenia is a severe mental disorder with established gender differences^1^ in the biological, psychological, and social presentation of the disorder.^2^ The risk of developing schizophrenia and having a more severe course of the disorder is higher among males compared with females,^1,3^ with females having fewer and shorter hospital admissions.^4–6^ The better course for females has been attributed to later onset, allowing increased social and occupational status.^7^ However, occasionally, females with schizophrenia present worse outcome data than males, as is the case with quality of life. Females report lower quality of life than males,^8^ including in global circumstances, health status and financial resources.^9^

The interplay between quality of life and the course of schizophrenia has been studied in the past. Higher quality of life is associated with lower levels of general psychopathology,^10^ less severe psychiatric symptoms^11^ and lower rates of psychiatric admissions.^12–14^ However, no single study to date has directly considered gender differences in the association between quality of life and the course of schizophrenia. The current study aims to examine gender differences in the association between quality of life and the risk of subsequent psychiatric admissions in a national sample with schizophrenia.

## Method

### Participants

The current study data were derived from the Psychiatric Rehabilitation Routine Outcome Measurement Project described elsewhere.^15^ All procedures contributing to this study were approved by and comply with the ethical standards of the Helsinki Committee at the Israeli Ministry of Health and the Institutional Review Board at the University of Haifa. Written informed consent was obtained from all participants. The study cohort comprised of participants with a last ICD-10 diagnosis of schizophrenia disorder (*n* = 1624), of whom 60.90% (*n* = 989) were males and 39.10% (*n* = 635) were females. This cohort received national psychiatric rehabilitation services in Israel. Participants with illicit drug addiction, violent behaviour or a lack of monitoring by a psychiatrist were not included in the study. Quality of life was assessed from 1 January 2013 to 19 August 2015.

### Additional data sources

Demographic data (i.e. gender), information on psychiatric hospital admissions (i.e. dates) and psychiatric diagnosis (i.e. ICD-10 diagnoses) were obtained from the Israeli National Psychiatric Case Registry. This registry contains lifelong listings of psychiatric admissions in Israel from 1950 onwards, with the accompanying updated ICD-10 diagnoses given by an Israeli medical board-certified psychiatrist. The diagnoses in this registry cover over 90% of persons with schizophrenia,^16^ are reported to be unchanged over time^17^ and have acceptable sensitivity compared with research diagnostic criteria.^18^ The National Psychiatric Case Registry identifies all patients admitted for broadly defined schizophrenia. The registry has been used in various studies.^19–21^

### Quality of life

Quality of life was measured using a translated version of the Manchester Short Assessment of Quality of Life, an abbreviation of the Lancashire Quality of Life Profile.^22^ Eight items measured satisfaction with physical and mental health, work or volunteering projects, social status, financial situation, family ties, leisure activities and residential status. The items were self-rated on a 5-point Likert scale. Higher scores marked higher quality of life. Research has shown that this measure has acceptable psychometric properties.^15^

### Analytic approach

Quality of life scores were summed based on the individual item scores. The course of schizophrenia was assessed based on subsequent psychiatric hospital admissions, meaning that admissions were analysed only if they occurred after the date that the quality of life questionnaire was completed. Participants were followed up for the risk of psychiatric admissions for 18 months or until their last psychiatric admission, whichever came first (as in prior studies^23^). To characterise the sample, descriptive statistics were computed. Next, the association between quality of life and the risk of psychiatric admissions was quantified using hazard ratios (HR) from Cox proportional hazards regression models.^24^ These models were computed for males and females separately, as well as for the entire cohort. All variables included in the regression models were checked for multicollinearity via the variance inflation factor for the entire cohort and the male and female subpopulations. Models were then computed unadjusted and adjusted for birth year and age at onset. Gender was an additional covariate in the model computed for the entire cohort. The proportional hazard assumption of the Cox regression models was tested by visualising Schoenfeld residual plots.^25^

All analyses were computed in R version 3.4.3 for windows using the ‘survival’ library^26^ for Cox proportional hazards regression models.

## Results

### Sample characteristics

The analytic sample was based on 1624 participants with schizophrenia, followed up for 18 months. Males constituted 60.90% of the total sample (*n* = 989) and females constituted 39.10% (*n* = 635). A subsample of 148 participants was admitted to a psychiatric hospital during the follow-up time. This subsample comprised 62.84% males (*n* = 93) and 37.16% females (*n* = 55). Both samples are characterised in Table 1.

**Table 1:** Sample characteristics for the entire cohort (*n* = 1624) and the subsample admitted to psychiatric hospitals during the 18-month follow-up (*n* = 148)

	Entire cohort – Males: *n* = 989; females: *n* = 635		Subsample admitted during follow-up – Males: *n* = 93; females: *n* = 55	
Variable	Mean (s.d.)	*P*	Mean (s.d.)	*P*
Quality of life score		0.29		0.77
Males	28.98 (5.56)		28.38 (6.41)	
Females	28.67 (5.64)		28.09 (5.14)	
Entire cohort/subsample	28.86 (5.59)		28.27 (5.95)	
Age at onset, years		<0.001		0.82
Males	26.12 (9.36)		25.26 (8.31)	
Females	27.96 (11.11)		24.93 (8.86)	
Entire cohort/subsample	26.84 (10.11)		25.14 (8.49)	
Birth year, years		<0.001		0.06
Males	1967 (12.15)		1970 (12.02)	
Females	1965 (12.31)		1966 (12.14)	
Entire cohort/subsample:	1967 (12.27)		1968 (12.17)	
Number of psychiatric admissions				
Males	–		1.67 (1.22)	0.88
Females			1.64 (1.14)	
Entire cohort/subsample			1.66 (1.69)	

### Quality of life and the risk of subsequent psychiatric admissions

The association between quality of life scores and the risk of subsequent psychiatric hospital admissions was quantified using hazard ratios from Cox proportional hazards models. All variables included in the models were checked for multicollinearity via the variance inflation factor and were found sufficiently uncorrelated (<1.5). For males, higher quality of life scores were statistically significantly (*P* < 0.05) associated with a reduced likelihood of subsequent psychiatric admissions (unadjusted: HR = 0.96, 95% CI 0.93–0.99; adjusted HR = 0.96, 95% CI 0.93–0.99). For females, there was a null association. For the entire cohort, higher quality of life scores were statistically significantly (*P* < 0.05) associated with a reduced likelihood of subsequent psychiatric hospital admissions (unadjusted: HR = 0.97, 95% CI 0.94–0.99; adjusted HR = 0.97, 95% CI 0.94–0.99), but no significant (*P* = 0.38) effect was found for gender. See Table 2 for all hazard ratios.

**Table 2:** Quality of life and the risk of subsequent psychiatric hospital admissions

	Hazard ratio (95% CI)	*P*
Males (*n* = 989)		
Quality of life (unadjusted)	0.96 (0.93–0.99)	0.04
Birth year	1.02 (0.99–1.04)	0.08
Age at onset	1.00 (0.97–1.02)	0.80
Quality of life (adjusted)	0.96 (0.93–0.99)	0.04
Females (*n* = 635)		
Quality of life (unadjusted)	0.97 (0.93–1.02)	0.38
Birth year	0.99 (0.97–1.02)	0.55
Age at onset	0.97 (0.94–0.99)	0.02
Quality of life (adjusted)	0.97 (0.93–1.02)	0.32
Entire Cohort (*n* = 1624)		
Quality of life (unadjusted)	0.97 (0.94–0.99)	0.04
Gender	0.85 (0.61–1.17)	0.32
Birth year	1.01 (0.99–1.02)	0.31
Age at onset	0.98 (0.97–1.00)	0.09
Quality of life (adjusted)	0.97 (0.94–0.99)	0.02

## Discussion

Based on data from a national sample with schizophrenia, the current study is the first to explore gender differences in the association between quality of life and the course of schizophrenia. Higher quality of life was associated with a lower risk of subsequent psychiatric hospital admissions among males but not among females.

The null association found for females may be explained by several tentative mechanisms, including the severity of psychiatric symptoms,^27^ oestrogen effects,^28^ social circumstances^29^ and premorbid functioning.^30^ Gender differences may be explained by combined biological and psychosocial factors^31^ and should be scrutinised in future research.

In schizophrenia research, gender differences are not always examined, although they are a consistently reported aspect of the disorder.^32^ Our results show that investigating gender differences in schizophrenia may identify otherwise masked patterns. The model we computed for the entire cohort did not reveal a significant effect for gender, unlike the analyses by gender separately. Furthermore, quality of life was significantly associated with hospital admissions for the entire sample, but within the female sample, the association was null. Exploration of gender differences may therefore have promising implications both in future research of schizophrenia and in clinical practice.^33^

### Limitations

The current study has notable limitations. Quality of life was assessed using the Manchester Short Assessment of Quality of Life.^22^ Broader measures that are tailored to schizophrenia (e.g. the Schizophrenia Quality of Life Scale^34^) exist that may yield different results. However, given the large sample of our study, the Manchester Short Assessment of Quality of Life was chosen for its quick and easy administration. Moreover, the measure has been used in multiple studies (e.g.^35^).

Our follow-up time was restricted to 18 months. This period exceeds the minimum 6-month follow-up that is considered adequate and exceeds the 12-month follow-up time in most studies.^36^ Nonetheless, if a longer follow-up time was used, the hospital admission rate would be greater and the results may have changed. A longer follow-up is therefore a direction for future research.

Finally, the current study did not account for all possible confounders that may influence the likelihood of psychiatric hospital admission, such as impaired global functioning, residual symptoms, adverse effects and medication non-adherence,^37^ or those that may specifically explain the null association found for females (e.g. oestrogen effects^28^). Age at onset was included as a confounder because it is established as associated with quality of life and psychiatric admissions in the literature.^6,38,39^ Future prospective studies are warranted to examine how additional factors influence the association between quality of life and psychiatric hospital admissions in both genders.

## Data Availability

The data that support the findings of this study are available from the corresponding author on reasonable request.
